# Identifying Household Level Risk Factors for Unintentional House Fire Incidents, Injuries and Fatalities

**DOI:** 10.23889/ijpds.v1i1.336

**Published:** 2017-04-19

**Authors:** Samantha Turner, Sarah Rodgers, Ronan Lyons

**Affiliations:** 1 Farr Institute, Swansea University

## Objective

Unintentional house fire incidents, injuries and deaths are a serious public health concern in the UK, which disproportionally affect certain groups in the population. Whilst house fires have decreased in recent years; growing financial pressures in the Fire and Rescue Services (FRSs) have resulted in funds dedicated to fire preventative activities becoming increasingly limited. To ensure ever limiting resources are targeted towards those households at greatest risk, it is essential the FRSs' are accurately informed about the types of household at increased risk. The aim of this project is to undertake a large-scale case-control study, to identify the distinguishing household level risk factors associated with unintentional house fire incidents, injuries and deaths. 

## Methods

Unintentional house fire incidents reported to the Welsh FRS between the years 2003-2008, were anonymised and incorporated into the Secure Anonymised Information Linkage (SAIL) Databank at the Farr Institute, Swansea University. 6943 case households (households which reported a fire to the FRS) were time-matched to 347,150 control households (case:control ratio 1:50). Individuals registered as living at these properties on the date of the fire were established using the Welsh Demographic Service (WDS) dataset. Household level variables will be created by linking case and control households to other demographic, health, educational and environmental datasets in SAIL. Conditional Logistic Regression will be used to estimate matched odds ratios and 95% confidence intervals.

## Results

Potential risk factor variables were selected on the basis of a systematic review and theoretically plausible variables. Covariates include: household composition (e.g. age and gender of residents), socioeconomic status, educational attainment, smoking, alcohol consumption, mental health conditions, other health related conditions, mobility and sensory impairments and property related characteristics. Fire related circumstances (e.g. fire ignition source, presence of a smoke alarm) will also be investigated in logistic regression models exploring risk factors for injury and death. Results will be presented at the conference. 

## Conclusion

This is the first large-scale analysis of risk factors for unintentional house fire incidents, injuries and deaths. The findings from this project will be translated into comprehensible infographics, designed to support the FRSs, other partner organisations and the general public, recognise high risk households in need of preventative interventions.

